# Systematic translation and adaptation of the FOCUS program, a USA-based supportive intervention for persons with cancer and their family caregivers, for use in six European countries

**DOI:** 10.1007/s00520-022-07391-x

**Published:** 2022-10-12

**Authors:** Maaike van der Wel, Doris van der Smissen, Sigrid Dierickx, Joachim Cohen, Peter Hudson, Aline De Vleminck, Lydia Tutt, David Scott, Silvia Di Leo, Caroline Moeller Arnfeldt, Catherine Jordan, Laurel Northouse, Judith Rietjens, Agnes van der Heide, Erica Witkamp

**Affiliations:** 1 Erasmus MC, University Medical Center Rotterdam, Rotterdam, the Netherlands; 2 End-of-Life Care Research Group, Ghent University and Vrije Universiteit Brussel, Ghent, Belgium; 3 End-of-Life Care Research Group, Vrije Universiteit Brussel (VUB) & Ghent University, Brussels, Belgium; 4 Centre for Palliative Care, St Vincents Hospital and The University of Melbourne, Melbourne, Australia; 5 Cicely Saunders Institute of Palliative Care, Policy and Rehabilitation, King’s College London, London, UK; 6 School of Nursing and Midwifery, The Queen’s University of Belfast, Belfast, UK; 7 Health Professions Department, Azienda USL-IRCCS Di Reggio Emilia, Reggio Emilia, Italy; 8 Department of Public Health, University of Copenhagen, Copenhagen, Denmark; 9 Palliative Care Research Unit, Department of Geriatrics and Palliative Medicine (GP), Bispebjerg and Frederiksberg Hospital, University of Copenhagen, Copenhagen, Denmark; 10 University College Dublin, National University of Ireland, Dublin, Ireland; 11 School of Nursing, University of Michigan, Ann Arbor, MI USA

**Keywords:** Translation and adaptation, Intervention, Supportive care, Family caregiver, Advanced cancer, PIPFLA

## Abstract

**Purpose:**

Having advanced cancer presents many challenges for patients and family caregivers. The FOCUS program is a psychoeducational nurse-led intervention, developed in the USA, to support dyads of patients with cancer and their family caregivers to live with the illness. The program includes a conversation manual and information resources for dyads. We aimed to develop a version of the program for dyads facing advanced cancer in six European countries.

**Method:**

The Participatory and Iterative Process Framework for Language Adaptation (PIPFLA) was used to guide the translation of the program to the local contexts of Belgium, Denmark, Ireland, Italy, the Netherlands, and the UK. In several rounds, potential program users (e.g., nurses, clinicians, patients, family caregivers) and researchers from all six countries reviewed program materials and advised on adaptations.

**Results:**

The PIPFLA process resulted in one European version of the program in different languages (FOCUS +). The FOCUS + conversation manual is uniform across all countries. The main adaptations included additional attention to both family caregiver and patient needs; more emphasis on self-management, advance care planning, and shared responsibilities; discussing the dyad’s outlook rather than optimism; addressing the role of nurses as educational rather than therapeutic; and more suggestions to refer dyads to health care professionals for specific care needs. The information resources for dyads were adapted to fit with local contexts.

**Conclusion:**

The PIPFLA methodology is an efficient and effective framework to thoroughly translate and culturally adapt a complex USA-based program for use in six European countries in collaboration with end users.

**Supplementary Information:**

The online version contains supplementary material available at 10.1007/s00520-022-07391-x.

## Introduction

Advanced cancer substantially affects the lives of patients and their relatives, with many relatives taking up a role as informal family caregiver [1, 2]. Family caregivers “provide physical, emotional, and instrumental support or assistance to individuals with a life-limiting illness that they view as family members” [3]. Family caregivers are likely to experience burden or anxiety [2, 4, 5]. Uncertainty and other consequences of advanced cancer affect the quality of life of both the patient and the family caregiver [1, 6–8]. Research indicates that the illness responses of cancer patients and family caregivers are interdependent; both mutually affect the other [9]. Health care professionals tend to focus mainly on relieving the symptoms of patients and may pay less attention to the needs of family [2]. Martire [10] found that dyadic interventions are more likely to have beneficial outcomes for both patients and their caregivers than approaching either of them separately. However, Matthys [11] reported a lack of evidence to help determine which psychosocial and educational interventions will generate the most favorable outcomes, for both patient and caregiver.

Several interventions have been developed to jointly support persons with advanced cancer and their family caregivers [12–15]. One of the most promising interventions is the FOCUS program, developed in the USA. FOCUS is a dyadic psychoeducational intervention designed to support cancer patients and their family caregivers in coping with the demands of the illness [16]. The intervention is based on the stress-appraisal model of Lazarus [17] that suggests that patients’ appraisal of and coping with their illness is influenced by personal, social, and illness-related factors [17, 18]. It is also based on the family stress theory, which implies that stress levels among family members are interdependent and that they mutually affect one another [19]. The FOCUS program consists of three structured nurse-led sessions with dyads, aimed at enhancing family engagement, optimistic attitude, effective coping strategies, dealing with uncertainty, and symptom management (i.e., F-O-C-U-S). Nurses delivering the program are trained extensively to understand its theoretical basis, in how to work with dyads, and in key psychoeducational communication skills. The nurse visits the dyad at home twice (sessions 1 and 3) and has one phone call meeting in between (session 2) [20]. The conversations are structured using a manual with a checklist format. The manual guides nurses in discussing several topics in a session with a dyad. Some examples of strategies for nurses to address the topics in a session are family engagement: encourage mutual support and teamwork; optimistic attitude: help dyads share fears and concerns; coping effectiveness: foster the use of active problem-focused coping strategies; uncertainty reduction: teach dyads how to be assertive to obtain additional information; and symptom management: teach self-care strategies to manage symptoms. The program also includes materials (i.e., leaflets and booklets) providing patients and family caregivers with additional information and resources.

Since the program’s start in 2002, it has been delivered to dyads in different regions in the USA facing different types and stages of cancer (breast, prostate, colorectal, lung). The program has been tested extensively with positive outcomes for both patients and their caregivers [20–22]. These positive outcomes were found in the areas of coping, self-efficacy, quality of life (social and emotional), emotional wellbeing, functional wellbeing, emotional distress, experiencing benefits of illness, uncertainty, communication, appraisal of caregiving, hopelessness, and symptom distress. The program’s effectiveness may be highly conditional and dependent on the national and cultural context in which patients and caregivers live, such as the availability of support and the cultural norms on family caregiving. It is likely that the USA and European countries have many variations in health-related norms, values, regulations, and care. In order to make the FOCUS program appropriate for use in Europe, a thoroughly conducted process of translation and adaption was needed to align it with the European context of oncology care and nursing [23].

## Method

The translation and adaptation of the FOCUS program was part of the DIAdIC project, a research program to evaluate Dyadic Psychoeducational Interventions for People with Advanced Cancer and their Informal Caregivers in an international randomized controlled trial. The countries involved in this study are Belgium, Denmark, Ireland, Italy, the Netherlands, and the UK (Northern Ireland and England). We used the Participatory and Iterative Process Framework for Language Adaptation (PIPFLA) to linguistically and culturally adapt the FOCUS program (23). PIPFLA was originally developed to translate and adapt an American-English intervention into an American-Hispanic intervention, using input from the perspectives of the intended users (i.e., the emic perspective, representing the local and within-culture perspectives of persons and professionals) and the perspectives of outsiders, such as researchers or politicians (i.e., the etic perspectives). The PIPFLA consists of multiple steps: (1) preparation, (2) forward translation, (3) backward translation, (4) review of backward translation, (5) harmonizing, (6) review by reference group (emic perspective), (7) harmonizing, (8) review by reference group (etic perspective), (9) harmonizing, (10) proofreading, and (11) final language adaptation [23].

Whereas the original PIPFLA approach assumes that a program is in principle suitable for the new context and starts with its linguistic translation, we started with a general assessment of the suitability of the program in each of the countries (Table 1). We extended the role of the reference groups. According to PIPFLA, the reference groups providing emic and etic perspectives advise once. We invited both reference groups to reflect on the program twice: they reviewed the program parallel to each other in the review phase, and their input was used equally in the harmonization phase. Forty-six people represented the emic perspective, and fifteen persons represented the etic perspective. A template was made to collect the feedback from all six countries. This template included questions about whether the conversation manual (i.e., intervention protocol manual) for the nurses was suitable to be used in the country concerned, and if not, what content should be changed, which items should be added, and which items could be left out. An overview was made of the feedback from all countries. This overview included recommended adaptations for each of the five items of FOCUS (family, outlook, coping, uncertainty, symptom management). The adaptations were discussed and processed step by step. The project team analyzed which content of the intervention had to be retained in order not to deviate too much from the theory underlying the original intervention. The original intervention designer (LN) was also consulted when considering adaptations of the manual for the nurses. Finally, the “TIDieR checklist (Template for Intervention Description and Replication),” was used to give a detailed description of the FOCUS + program (Appendix) [24]. The translation and adaptation process was undertaken from May 2019 to December 2020.

**Table 1:** Adapted PIPFLA steps to linguistically and culturally translate the FOCUS program

Steps	Activities
1: Preparation	Each country establishes a reference group for etic and emic perspectives Each country appoints a national adaptation and translation coordinator (NATC) to represent the country in the international process Each (non-English speaking) country appoints 2 forward and 2 backward translators
2: Review	
2a: Etic perspective	National reference groups of researchers review original materials in English and identify where adaptation may be needed
2b: Emic perspective	National reference groups of end users (nurses, clinicians, patients, family caregivers) review original materials in English and identify where adaptation may be needed
2c: Summarize and report	NATCs report findings from reference groups to an international coordinator
3: Harmonize	International coordinator reviews NATC reports and decides together with NATCs about one common version of the materials in English to be translated
4: Forward translation	National forward translators translate materials from English into target languages
5: Backward translation and validation	National backward translators translate materials from target languages into English NATCs compare backward-translated materials to original English materials, identify discrepancies, and decide about how to resolve these
6: Review	
6a: Etic perspective	National reference groups of researchers review materials in local language and identify where adaptation may be needed
6b: Emic perspective	National reference groups of end users (nurses, clinicians, patients, family caregivers) review materials in local language and identify where adaptation may be needed
7: Harmonize	NATCs review findings from reference groups and decide how to address them in materials in local language
8: Review	Coordinator, NATCs, and original developer discuss all national deviations and reach consensus about the final version of the materials
9: Proofread and complete process	NATCs proofread final versions and make final language corrections

## Results

All steps of the translation and adaptation process were followed as planned, resulting in an adapted version of the FOCUS program: the FOCUS + program. The FOCUS + conversation manual for nurses is uniform across all countries, with country-specific aspects in terms of language. For sensitive topics, such as the end of life and sexuality, slight deviations from the original wording were allowed if these were considered more appropriate. The additional information materials and resources for the dyads are largely country-specific and adapted to national healthcare systems and regulations.

### *FOCUS* + *manual for nurses*

Adaptations to the original FOCUS manual address seven themes or areas. Specific examples of adaptations are included in Table 2.

1. Needs and wishes of the family caregiver
  In the original FOCUS program, the family caregiver seems primarily positioned as a person supporting the patient. Reference groups of researchers and users advised putting more emphasis on the impact of the patient’s illness on family caregivers themselves. In FOCUS + , the values and needs of both individuals within the dyad are addressed equally. Questions about family concerns were added, and questions focusing primarily on the patient in the original FOCUS program were revised and directed towards the dyad.
2. Self-management
  Several items in the original FOCUS program were considered to be too normative or prescriptive in the local culture of the six countries, such as the sentence instructing the nurse to “teach (the dyad) about benefits of active versus passive coping strategies.” Furthermore, a normative or prescriptive approach was considered not to comply with current views on the enhancement of self-management. Members of reference groups in several countries suggested that, whereas self-management at the beginning of this century focused on treatment compliance, it is nowadays, and in the context of advanced cancer, seen as more comprehensively “managing the physical, psychosocial and existential consequences of living with a progressive and life-threatening disease and its treatment” [7]. As a result, some parts from the original FOCUS program were adapted in the European FOCUS + program to promote open and equal communication between the nurse and the dyad and to more explicitly support self-management strategies of the dyad.
3. The concept of optimism
  The emphasis on “optimism” as the preferred mindset in the FOCUS program was considered too normative in the context of advanced cancer by most of the reference groups. According to the reference groups, the original approach seemed to suggest that it is best for dyads to be optimistic, where it may also be beneficial to acknowledge and discuss negative or pessimistic feelings of the patient, the family caregiver, or both. Therefore, the reference groups recommended the use of a more neutral approach to give dyads the opportunity to discuss both positive and negative feelings. “Outlook” was considered to be a more neutral term and a better fit with the European context. Therefore, the term “Optimism” in the original FOCUS program was changed to “Outlook” in the European FOCUS + program. The item “optimism assessment” was changed into “assess the dyad’s outlook about their situation.”
4. The supportive and educational character of the program
  According to several reference groups, some wording in the original FOCUS manual seemed to have “therapeutic” or psychological care-oriented intentions. Psychological interventions were considered not to be a formal responsibility of nurses in the European countries involved, which may be different from the role of (oncology) nurses in the USA. A consensus was reached that the goal of the FOCUS + program should be framed as improving the dyad’s self-management and self-efficacy by providing information and psychoeducation. Adaptations were made to emphasize the supportive and educational nature of the nursing intervention.
5. The order of topic
  The reference groups recommended changes in the order of the topics in the manual. For example, discussing concerns about children was part of the third session of the original FOCUS program. This topic was moved to the first session in the FOCUS + program, because reference groups stated that it is a priority for many patients and family caregivers. Another example is the topic of intimacy and sexuality. In the original FOCUS program, this topic is discussed in the second meeting, which is conducted by telephone. Discussing a potentially sensitive topic such as intimacy and sexuality over the phone was considered inappropriate. In the European program, this topic is therefore discussed in the second face-to-face meeting at the patient’s home.
6. Referral to health care professionals

**Table 2:** Examples of adaptations in FOCUS + conversation manual for nurses

Theme	Wording in original FOCUS conversation manual	Adapted wording in FOCUS + conversation manual
Needs and wishes of family caregiver	“Follow-up with patient about symptoms he/she is experiencing.” Example from session 2	“Follow-up with patient about symptoms he/she is experiencing Ask if the caregiver has experienced any symptoms since last session.” Example from session 2
Self-management	“Teach about benefits of active versus passive coping strategies. Active (effective/healthy): Problem-solving, seeking help, finding support. Passive (avoidant/unhealthy): Use of alcohol, distancing from caregiver/family/friends, total denial.” Example from session 1	“Discuss different coping strategies. Explain by giving examples of active (e.g., goal setting) and passive coping (e.g., denial). Ask which (active) coping strategies have been effective in the past and encourage them to change their current strategies to these more effective ones.” Example from session 1
The concept of optimism	“Introduce importance and benefits of optimism.” Example from session 1	“Ask the patient and the caregiver how they deal with (positive and negative) feelings and thoughts.” Example from session 1
Supportive and educational character	“Establish a therapeutic alliance” Example from session 1	“Establish an educative alliance” Example from session 1
Order of topics	“Discussing concerns about children is part of session 3” Example from session 3	“Discussing concerns about children is part of session 1 and 3” Example from session 1 and 3
Referral to health care professionals	“Clarify information on patient’s specific treatment including chemotherapy (e.g., paclitaxel, etoposide, etc.), radiation, and hormonal therapies (e.g., leuprolide, goserelin, tamoxifen, etc.). Name/classification of drugs, side effects, when to notify doctor, etc. Give handout on each drug or treatment as needed.” Example from session 1	“If needed refer to a health professional (e.g., physician), leaflets or website for further information about treatment and drug. If applicable, offer dyad the appropriate information from the FOCUS + Guide: Chapter x, question x” Example from session 1

Both FOCUS and FOCUS + include referrals to additional information resources where this may be helpful. In case a dyad has specific questions about the diagnosis and treatment options, the intervention nurse is expected to provide such information in the original program. In FOCUS + , dyads are referred to their attending health care professionals. This decision was made because the complexity of current individualized and targeted therapies in oncology treatment may exceed the knowledge and competencies on FOCUS + intervention nurses.

### *FOCUS* + *additional materials for dyads*

In addition to the manual for nurses, the original FOCUS program includes a wide collection of additional information resources, such as leaflets about therapies, booklets on physical, social, psychosocial, or spiritual consequences of cancer, and websites to various American guidelines and resources. Because each country was expected to have many equivalents of the materials, translation of all original materials was judged not to be necessary. In addition, original American materials often did not comply with the diversity of healthcare services and support in Europe.

Therefore, each country selected national equivalents of the original USA leaflets and booklets, with an even wider range than the original FOCUS materials in terms of content, format, and application level, varying from hospital-based to national guidelines. During the evaluation of the national equivalent materials, some of them were updated because of the availability of new treatment or new communication options.

The project management team recommended integrating all information resources into one booklet. This booklet was developed by the project management team and follows the structure and content of the conversation manual so that it can serve as a reference book for dyads in all stages of the intervention and beyond. The booklet was tailored to the dyad, e.g., by including exercises about dyadic communication.

## Discussion

This paper presents the systematic translation and cultural adaptation of a psychoeducational program, originally developed and evaluated in the USA for dyads of cancer patients and family caregivers, to a European dyadic program for patients with advanced cancer and family caregivers. The main themes of adaptations of the FOCUS + manual for nurses were the needs and wishes of the family caregiver; self-management; the concept of optimism; the supportive and educational character of the program; the order of topics; and referral to health care professionals. The additional information resources for dyads were adapted to local contexts.

The PIPFLA was found to be a useful framework to develop a program that fits into the living context and treatment of the target group while remaining faithful to the original program. The main feature of PIPFLA is the structured participation of various stakeholders in the translation process, ensuring input from the perspectives of potential end users (“emic”) and scientific experts (“etic”). Using these perspectives together may cause tension, for instance, when negotiation is needed to resolve differences between end users’ preferences and scientific guidelines. This usually does not outweigh the benefit of incorporating both perspectives [25]. In our study, all six countries provided “emic” and “etic” perspectives on the translations and adaptations. Combining both perspectives generally turned out to enrich discussions about the tone, content, and order of the program topics.

As the six European countries differ in language, culture, and the organization of healthcare, the translation and adaptation process took time, negotiation, and compromise. For example, addressing “end of life” issues requires nurses to take a careful and empathetic approach with dyads. Both patients’ and family caregivers’ perspectives in each country made a meaningful contribution to discussions about this item. In their opinion, it is important that the nurse gives dyads the opportunity to talk about the end of life and that the nurse would do this sensitively using a more indirect approach. Nurses from each country would use local terminology which is appropriate for this sensitive approach. There were also topics on which all reference groups agreed, such as replacing the concept of optimism to outlook. This example took less time to discuss.

The PIPFLA aligns with ADAPT guidance on adapting interventions to new contexts. ADAPT is an update of the well-known Medical Research Council guidance, published in November 2020 [26]. The ADAPT guidance suggests that the “use of interventions with a previous evidence base in new contexts might be more efficient than developing new interventions.” ADAPT guidance emphasizes the importance of a structured process of adaptation and of involvement of stakeholders, in case an intervention has content that is highly sensitive to cultural context. Furthermore, it stresses the importance of reporting the adaptation processes and outcomes. The PIPFLA turned out to be a feasible framework to structure the translation and adaptation process in collaboration with nurses, clinicians, patients, caregivers, and researchers, while taking into account the contextual differences in cancer care in America vs. Europe. We argue that when setting the reuse of interventions as a norm, and interventions will increasingly be translated, such a framework is required to guide the process, similar to guidelines for scientific linguistic translation of questionnaires.

The European FOCUS + program will be evaluated in an international randomized controlled trial. Studying the effectiveness of a complex psychoeducational intervention requires standardization. However, complex interventions may work best when tailored to local circumstances rather than being completely standardized [27, 28]. We, therefore, allowed some cross-country variation in materials, such as leaflets or websites dyads may be referred to. A careful process evaluation of cross-country variance in the implementation of the program and in nurses’ fidelity to its structure and content is needed.

### Implications for practice

Although nurses are increasingly aware of the needs of patients and their relatives, their approach to identifying and discussing those needs is typically unstructured with supportive tools lacking [29, 30]. The FOCUS + intervention provides nurses an evidence-based method to give structured support to both patients and caregivers, to help them deal with the illness and its impact on their personal situation. With FOCUS + dyads of patients and caregivers are offered the opportunity to discuss their concerns, to obtain additional information, and to learn new ways to manage their concerns. This may in the end benefit dyad’s mutual communication and self-efficacy.

## Conclusion

Transforming the American nurse-led FOCUS program for persons with advanced cancer and their family caregivers to a European FOCUS + version to be implemented in six countries (Belgium, Denmark, Ireland Italy, the Netherlands, and the UK) required a series of country-specific adaptations. Our study shows that the PIPFLA methodology provided an efficient and effective framework for the translation and adaption of such a complex psychoeducational intervention. PIPFLA enabled us to make cultural and linguistic adjustments together with the end users, while staying close to the original program.

## Supplementary information

Below is the link to the electronic supplementary material.

Supplementary file1 (DOCX 20.1 KB)

## Data Availability

Not applicable.
