# Supplementary material for: Systematic translation and adaptation of the FOCUS program, a USA-based supportive intervention for persons with cancer and their family caregivers, for use in six European countries
Source: Support Care Cancer. 2022 Oct 12;30(12):9763–70. doi: 10.1007/s00520-022-07391-x (PMC9715502; doi:10.1007/s00520-022-07391-x)
Supplement: Supplementary file 1 — Supplementary file1 (DOCX 20.1 KB) [file 520_2022_7391_MOESM1_ESM.docx]

**Additional 1**

*Table 3 FOCUS+ program*

| **FOCUS+** |
| --- |
| FOCUS+ program: Family involvement, Outlook, Coping, Uncertainty, Symptom management. A psycho-educational nurse-led face-to-face intervention to support patients with advanced cancer and their caregiver together. |
| **WHY** |
| The FOCUS+ program is a multi-component, psychoeducational, dyadic intervention, tailored to the specific needs of the dyad that is aimed at improving the quality of life and well-being of patients with advanced cancer and their family caregivers. Specific aims are:  1. Improve dyad’s mutual communication  2. Identify dyads’ identity and meaning-making  3. Increase dyads’ coping skills  4. Improve dyads’ self-efficacy |
| **WHAT** |
| The FOCUS+ program consists of five core content areas, with the first letter of each area forming the acronym FOCUS: 1) Family involvement, 2) Outlook, 3) Coping effectiveness, 4) Uncertainty reduction, and 5) Symptom management. Each of the five core content areas were derived from prior research.  A 17-page conversation manual outlines the intervention in a checklist format. The program has flexibility to tailor its content to the needs of specific family dyads. For example, some dyads may mainly need assistance with symptom management, while others mainly need assistance with psychosocial issues.  The intervention is delivered by a nurse, who uses the conversation manual during three subsequent meetings with the dyad. All FOCUS+ items (F, O, C, U, S) are discussed in every session. The manual includes references to leaflets or websites where the dyad can find additional information or support. The nurse tailors the intervention to the needs of the dyad and supports the dyad in discussing items that are important to them. A printed booklet that provides an overview of the three sessions as well guidance to additional information resources is handed out to the dyad before the first session. This core booklet serves as a reference guide for the dyads to be used at their own discretion and reinforces key messages linked to the core components of the intervention. |
| **PROVIDER** |
| Intervention nurses are key to the success of the FOCUS+ program through their ability to build trustful relationships with the patient-carer dyad and by implementing the intervention consistently and with empathy. Nurses are trained in delivering the intervention during a 3-day training program. |
| **HOW** |
| The intervention consists of 3 sessions over a 12-week period. The first and third sessions are face-to-face meetings of the nurse and the dyad at the home of the dyad (or at a location of the dyad’s preference). The second session consists of a telephone or video call of the nurse and the dyad. |
| **WHERE** |
| Face-to-face sessions will take place at the home of the dyad (or at a location of the dyad’s preference). |
| **WHEN and HOW MUCH** |
| The intervention consists of 3 sessions over a 12-week period. The first session takes about 90 minutes. After 4-5 weeks the second session is scheduled which takes about 30 minutes. The third session is scheduled at week 9-10 and lasts about 90 minutes. |
| **TAILORING** |
| The nurse discusses all the FOCUS+ elements at each session, using the intervention manual. In addition, the nurse discusses other needs of the patient and caregiver. Depending on the dyad's situation and the personal needs of both, a wide variety of problems can be addressed. If there is a need for support, the nurse either provides such support or refers the dyad to appropriate health care professionals, organizations or information sources. When additional personal problems of the dyad are discussed during the first session, the nurse makes notes to ensure that these problems are evaluated during the second and third session. |

# Article title: Systematic translation and adaptation of the FOCUS program, a USA based supportive intervention for persons with cancer and their family caregivers, for use in six European countries

Journal name: Supportive Care in Cancer
Author names: Maaike van der Wel¹, Doris van der Smissen¹, Sigrid Dierickx², Joachim Cohen³, Peter Hudson³ ¹⁰, Aline De Vleminck³, Lydia Tutt ⁴, David Scott ⁵, Silvia Di Leo⁶, Caroline Moeller Arnfeldt⁷, Catherine Jordan⁸, Laurel Northouse⁹, Judith Rietjens¹, Agnes van der Heide¹, Erica Witkamp¹, on behalf of DIAdIC
Affiliation and e-mail address of the corresponding author: Maaike van der Wel , Erasmus MC, University Medical Center Rotterdam, the Netherlands, m.vanderwel.1@erasmusmc.n
